# Innovative Extraction Techniques and Hyphenated Instrument Configuration for Complex Matrices Analysis

**DOI:** 10.3390/molecules23092391

**Published:** 2018-09-18

**Authors:** Marcello Locatelli, Simone Carradori, Andrei Mocan

**Affiliations:** 1 Department of Pharmacy, University “G. d’Annunzio” of Chieti-Pescara, Via dei Vestini 31, 66100 Chieti, Italy; 2 Department of Pharmaceutical Botany, Faculty of Pharmacy, “Iuliu Haţieganu” University of Medicine and Pharmacy, 400337 Cluj-Napoca, Romania

This special issue was proposed by three Co-Guest-Editors with complementary expertise in the fields of Analytical Chemistry, Medicinal Chemistry, and Pharmaceutical Botany to better understand the most recent techniques to extract, isolate, characterize, and biologically evaluate natural occurring compounds from complex matrices (plant extracts, biological fluids). The interest in this research field is demonstrated by relevant literature in high impact factor journals such as Molecules (http://www.mdpi.com/journal/molecules/special_issues), which promoted this special issue with an emphasis on the most innovative approaches to the matter.

The complexity of the topic requires knowledge of analytical chemistry, extraction procedures, validation of statistical approaches, botany, and chemical/enzymatic stability of natural compounds. We selected 18 manuscripts (one review and 17 research articles) submitted by researchers from different countries that fit the aims and scope of our mission. We are also grateful to all the contributors and colleagues/reviewers who devoted their precious time and expertise to finalize this special issue. Lastly, we want to thank MDPI publisher and the Editorial staff of the journal for their constant and professional support.

Samanidou’s research group, who are strongly involved in the development of innovative extraction analyses under a rigorous validation method, described exhaustively the “state of the art” of Ionic Liquids (ILs) in the extraction procedures [1]. Pros and cons were considered and justified the role of ILs in miniaturized microextraction techniques, such as solid-phase microextraction (SPME), dispersive liquid-liquid microextraction (DLLME), single-drop microextraction (SDME), stir bar sorptive extraction (SBSE), and stir cake sorptive extraction (SCSE). The versatility of ILs, beyond their use as extraction solvents, is characterized by the evidence that they could provide alternative advantages as intermediate solvents, mediators, and desorption solvents [2].

The other 17 research articles can be divided into three main groups.

The first one is related to the application of validated methods for the detection and quantification of drugs or metabolites in real samples/complex matrices. Panderi et al. [3] studied an accurate and precise determination of metformin and rosuvastatin in human plasma by HILIC-ESI/MS (Hydrophilic Interaction Liquid Chromatography-Electrospray Ionization Mass Spectrometry), limiting the sample preparation process and the chromatographic run time. These procedures were also applied for their suitability in the routine analysis of plasma samples from eight patients under this therapeutic treatment. He et al. [4] reported the determination by High Performance Liquid Chromatography-Quadrupole Time-of-Flight Mass Spectrometry (HPLC-Q-TOF-MS) using three important branched-chain ketoacids (α-ketoisocaproate, α-keto-β-methylvalerate and α-ketoisovalerate) in serum and muscle samples.

The second group of articles dealt with the application of innovative analytical techniques for environmental purposes. Huang et al. [5] used GC-MS and GC-O (Gas Chromatography-Mass Spectrometry/Olfactometry) for the identification of volatile compounds as an attempt to monitor indoor air quality. Zhenh et al. [6] proposed a daily monitoring of yttrium and rare earth elements (YREEs) in seawater by ICP-MS (Inductively Coupled Plasma-Mass Spectrometry) coupled to a cheap flow injection system online and to a specific pre-concentration step.

The third group of research articles analyzed plant and food matrices, characterized by a high economic, ethnopharmacological, and health-promoting value. The first three articles [7,8,9] tried to better understand the parameters influencing the extraction of polyphenols, alkaloids, and gelatin from natural sources. The authors compared and implemented their procedures by adding enzymes (actinidin) or specific substances (magnetite). Other important papers explore exhaustively by means of innovative equipment such as UPLC-MS (Ultra Performance Liquid Chromatography-Mass Spectrometry) [10], HSCCC (High-Speed Counter-Current Chromatography) [11], NIR (Near Infrared spectroscopy) [12], and UPLC-qTOF MS/UPLC-QqQ MS [13,14] plants and their derived products. Lastly, some research articles were devoted not only to the recovery and full characterization of plant metabolites, but also to the assessment of their biological activity against a panel of pharmacologically relevant targets (acetylcholinesterase, tyrosinase, α-amylase, sirtuin 1, hematopoiesis and hemostasis, skin-whitening ability) [15,16,17,18,19].
